# In-vitro evaluation of different antimicrobial combinations with and without colistin against carbapenem-resistant *Acinetobacter baumannii* clinical isolates

**DOI:** 10.1186/s40001-024-01885-6

**Published:** 2024-06-16

**Authors:** Tuba Müderris, Gülden Dursun Manyaslı, Nurbanu Sezak, Selçuk Kaya, Tuna Demirdal, Süreyya Gül Yurtsever

**Affiliations:** 1 Faculty of Medicine, Department of Medical Microbiology, İzmir Katip Çelebi University, İzmir, Türkiye; 2 Cizre Dr. Selahattin Cizrelioğlu Public Hospital, Department of Medical Microbiology, Şırnak, Türkiye; 3 Faculty of Medicine, Department of Infectious Diseases and Clinical Microbiology, İzmir Demokrasi University, İzmir, Türkiye; 4 Faculty of Medicine, Department of Infectious Diseases and Clinical Microbiology, İzmir Katip Çelebi University, İzmir, Türkiye

**Keywords:** In vitro antimicrobial interactions, Carbapenem resistance *Acinetobacter baumannii*, Combined antibiotic therapy, Checkerboard assays, Epsilometer test synergy assays

## Abstract

**Background:**

Carbapenem-resistant *Acinetobacter baumannii* (CRAB) infections are one of the most common causes of nosocomial infections and have high mortality rates due to difficulties in treatment. In this study, the in vitro synergistic interactions of the colistin (CT)–meropenem (MEM) combination and patient clinical outcomes were compared in CRAB-infected patients that receive CT–MEM antimicrobial combination therapy. In addition, in vitro synergistic interactions of MEM–ertapenem (ETP), MEM–fosfomycin (FF) and CT–FF antimicrobial combinations were investigated. Finally, the epsilometer (*E*) test and checkerboard test results were compared and the compatibility of these two tests was evaluated.

**Methods:**

Twenty-one patients were included in the study. Bacterial identification was performed with MALDI–TOF, and antimicrobial susceptibility was assessed with an automated system. Synergy studies were performed using the *E* test and checkerboard method.

**Results:**

For the checkerboard method, the synergy rates for CT–MEM, MEM–FF, MEM–ETP and CT–FF were 100%, 52.3%, 23.8% and 28.5%, respectively. In the *E* test synergy tests, synergistic effects were detected for two isolates each in the CT–MEM and CT–FF combinations. Microbial eradication was achieved in nine (52.9%) of the 17 patients that received CT–MEM combination therapy. The agreement between the *E* test and the checkerboard test was 6.5%.

**Conclusions:**

A synergistic effect was found with the checkerboard method for the CT–MEM combination in all isolates in our study, and approximately 70% of the patients benefited from treatment with this combination. In addition, more than half of the isolates showed a synergistic effect for the MEM–FF combination. Combinations of CT–MEM and MEM–FF may be options for the treatment of CRAB infections. However, a comprehensive understanding of the potential of the microorganism to develop resistant mutants under applied exposures, as well as factors that directly affect antimicrobial activity, such as pharmacokinetics/pharmacodynamics, is essential for providing treatment advice. We found a low rate of agreement between the *E* test method and the checkerboard test method in our study, in contrast to the literature. Comprehensive studies that compare clinical results with methods are needed to determine the ideal synergy test and interpretation method.

## Background

Carbapenem-resistant *Acinetobacter baumannii* (CRAB) remains one of the most important public health problems of the twenty-first century and is among the top priority pathogens for which new antibiotics are needed to be developed[1]. These bacteria are especially known for their ability to survive in hospital environments, evade host immunity, and acquire new antibiotic resistance mechanisms [1]. No treatment has been shown to significantly reduce mortality in patients with CRAB infections [2, 3], and 28-day mortality rates have been reported to exceed 45% [1].

The agents showing the highest in vitro activity against these isolates are polymyxins, tetracyclines and β-lactams [1]. Both the Infectious Diseases Society of America Antimicrobial Resistance Treatment Guideline and the European Society for Clinical Microbiology and Infectious Diseases Antimicrobial Resistance Treatment Guideline recommend combination therapy with at least two in vitro active agents for serious CRAB infections [4, 5]. According to data collected worldwide, colistin (CT) forms the backbone of combination therapy [6–10]. While the limitations of CT are well-known [11], the rationale for using colistin with carbapenem has been confirmed by high in vitro synergy rates in multiple studies [12–14]. However, considering the mortality rates, the superiority of combination therapy of CT with carbapenem over CT therapy alone is controversial [3].

In our hospital, CT–meropenem (MEM)-based combinations are preferred as the first choice for the treatment of CRAB infections. In our study, the in vitro synergistic interactions of CT–MEM combination and patient clinical outcomes were compared in patients with CRAB infection who started treatment with the CT–MEM antimicrobial combination. In addition, this study was aimed at determining alternative treatment options by revealing the in vitro synergistic interactions of MEM–ertapenem (ETP), MEM–fosfomycin (FF) and CT–FF antimicrobial combinations. The results of the epsilometer (E) synergy assay, which is superior to other in vitro synergy tests in terms of ease of use, and checkerboard test results are compared.

## Methods

### Patient selection

Twenty-one patients who were hospitalized in the intensive care units of İzmir Atatürk education and research hospital, whose CRAB isolates were recovered from various samples and for whom combined antimicrobial therapy was started, were included in this study. Patients with polymicrobials and multiple sources of infection were not included in the study. The source of infection was determined according to the diagnostic criteria of the Centers for Disease Control and Prevention [15]. The clinical information of patients included in the study was reviewed.

### Definitions

Preantibiotic use was defined as the use of antibiotics for at least 72 h in the 30 day period before the first microbiological infection diagnosis. Clinical outcomes were defined as follows: microbial eradication—absence of growth in the control culture performed on the 10th day of antimicrobial therapy; cure—clinical improvement and culture negative following treatment; clinical improvement—normal body temperature, normal level of white blood cells and stable vital signs without microbiologic confirmation of a cure; treatment failure—worsening clinical symptoms or the requirement for different or additional antimicrobial therapy against CRAB infection; and death in hospital following CRAB infection.

### Identification and antimicrobial susceptibility

All the isolates were identified using matrix-assisted laser desorption ionization–time of flight (MALDI–TOF) (Bruker, BD, USA). Antimicrobial susceptibility was assessed using an automated system (Phoenix, BD, USA). The results were evaluated according to the European Committee on Antimicrobial Susceptibility Testing (EUCAST) guidelines [16]. The minimum inhibitory concentrations (MICs) were determined using the broth dilution method for MEM, CT and ETP, and the agar dilution method for FF [16].

### Synergy studies

#### Checkerboard assays

CT sulfate (Carbosynth, USA), MEM trihydrate (Chem-Impex, USA), ETP sodium (Carbosynth, USA) and FF (Koçak Farma, Turkey) were prepared as stock solutions with concentrations of 4096 µg/ml. The bacterial suspension was prepared, so that the final bacterial concentration was approximately 5 × 10^5^ CFU/ml. When testing combinations containing FF, 25 µg/mL of glucose 6 phosphate was added to the medium. The antimicrobial concentration range for each isolate was calculated as 0.031xMIC–4xMIC. It was performed as described in the literature [17].

The fractional inhibitory concentration index (FICI) was calculated for each antibiotic in each combination by using the following formula: FICA + FICB = FICI, where FICA denotes the MIC of drug A in combination divided by the MIC of drug A alone and FICB represents the MIC of drug B in combination divided by the MIC of drug B alone. The FICIs were interpreted as follows: ≤ 0.5, > 0.5– ≤ 1.0, > 1.0– ≤ 4.0 and > 4.0 were interpreted as synergistic, additive, indifferent, and antagonistic effects, respectively [17, 18].

#### E synergy assays

After the bacterial suspension was prepared at a 0.5 McFarland concentration on Mueller–Hinton agar medium, it was spread homogeneously on the entire surface of the medium using a swab, as previously described [18].

Drug A and Drug B *E* test strips were placed on different sections of the Mueller–Hinton agar (MHA) plate. The agar was marked with an inoculating loop adjacent to the previously determined MIC value on each strip. For isolates where the MIC exceeded the concentration on the *E* test strip, the highest concentration was marked on the agar. Determination of the MIC value of Drug A in the combination of Drug A and Drug B; Drug A strips were removed and discarded after 1 h of incubation at room temperature. Drug B was placed on the area of the previously removed strip, so that the drug B MIC corresponded with the mark of the drug A MIC. The results for both antimicrobials were read after 16–20 h incubation in ambient air at 35 °C. The same application was made for the drug B strip placed on the side. The test results were calculated and interpreted as in the checkerboard test.

*A. baumannii* ATCC 19606 standard strain was used to check whether the antibiotic taken from the *E* test strip sufficiently diffused into the agar within 1 h. After the standard strain was plated on Mueller–Hinton agar medium, the *E* test strip was placed and the strip was removed after 1 h. The MIC value obtained after the plate was incubated at 35 °C for 18 h was compared with the MIC value obtained by the microdilution method. If the same result was obtained with both methods, the antibiotic was considered to have sufficiently diffused into the agar in 1 h at room temperature.

## Results

### Patient characteristics

Of the 21 patients with CRAB isolate infections, 11 patients were male, and the median age was 74 years. The most common causes of comorbidities were cerebrovascular disease, cancer, diabetes mellitus, hypertension and renal failure. The sources of infection were hospital-acquired pneumonia (*n* = 18) and bloodstream infection (*n *= 3). Thirteen of the patients developed septic shock. Three patients had previously used colistin, and eleven had previously used carbapenem. All patients had undergone invasive intervention before infection. Six patients had undergone surgery before infection (Table 1).

**Table 1:** Characteristics of patients infected with *Acinetobacter baumannii*

Patient Number	Age (Years)	Sex	Comorbidity	Diagnosis	Septic shock	Prior colistin use	Prior carbapenem use	İnvasive intervention	Operation
**1**	70	M	CVD	BSI	Yes	No	No	Yes	No
**2**	83	F	CVD	HAP	Yes	No	No	Yes	No
**3**	52	F	CA	HAP	No	No	Yes	Yes	Yes
**4**	85	M	CVD	HAP	No	No	No	Yes	No
**5**	67	M	GIB, CA	HAP	Yes	No	Yes	Yes	No
**6**	74	F	DM, HT, E	HAP	Yes	No	Yes	Yes	No
**7**	81	M	RF	HAP	Yes	No	Yes	Yes	No
**8**	72	F	RF, PE, SE	HAP	Yes	No	Yes	Yes	No
**9**	79	M	CVD	HAP	No	No	No	Yes	No
**10**	78	F	CA	HAP	Yes	No	No	Yes	No
**11**	80	F	CHF, HT, RF, CVD, PEM, CA	BSI	Yes	No	No	Yes	Yes
**12**	60	M	-	HAP	No	No	Yes	Yes	No
**13**	78	M	CVD, HD	HAP	Yes	No	No	Yes	No
**14**	25	F	SE	BSI	Yes	Yes	Yes	Yes	No
**15**	72	M	CVD	HAP	No	Yes	Yes	Yes	Yes
**16**	69	M	CHF, COPD, CA	HAP	Yes	No	Yes	Yes	No
**17**	50	F	CAR, DM, CVD	HAP	Yes	No	Yes	Yes	Yes
**18**	75	M	DM, HT, CVD	HAP	No	Yes	Yes	Yes	Yes
**19**	79	M	CVD	HAP	No	No	No	Yes	No
**20**	75	F	CVD	HAP	Yes	No	No	Yes	Yes
**21**	53	F	CVD	HAP	No	No	No	Yes	No

CVD, cerebrovascular disease; CA, cancer; GIB, gastrointestinal bleeding; DM, diabetes mellitus; HT, hypertension; E, epilepsy; RF, renal failure; PE, pulmonary embolism; SE, status epilepticus; CHF, congestive heart failure; PEM, pulmonary edema; HD, hemodialysis; COPD, chronic obstructive pulmonary disease; CAR, cerebellar aneurysma rupturing; BSI, bloodstream infection; HAP, hospital-acquired pneumonia

### Antimicrobial susceptibility

Six of the strains included in the study were isolated from blood, and the other strains were isolated from tracheal aspirate samples (Table 2). The MIC distributions of the antimicrobial agents are detailed in Table 2. All the isolates were resistant to amikacin, ciprofloxacin, gentamicin, imipenem, levofloxacin and MEM. In addition, two isolates were found to be resistant to CT.

**Table 2:** Clinical samples from which isolates were isolated and MICs of antimicrobials

Patient Number		MIC (mg/L)									
	Sample	AK	CIP	CN	SXT	IMP	LEV	MEM	ETP	FF	CT
1	Blood	> 16	> 1	> 4	≤ 2	> 8	-	32	32	128	0.25
2	Blood	> 16	> 2	> 4	≤ 1	> 8	-	32	32	128	0.25
3	TA	> 16	> 2	> 4	-	> 8	-	32	32	256	0.25
4	TA	> 16	> 2	> 4	≤ 1	> 8	-	32	32	64	1
5	Blood	> 16	> 2	> 4	≤ 4	> 8	-	32	32	128	0.25
6	TA	> 32	> 1	> 8	≤ 8	> 8	> 2	32	32	256	0.25
7	TA	> 32	> 1	> 8	≤ 8	> 8	> 2	32	32	256	0.25
8	TA	> 32	> 1	> 8	≤ 2	> 8	> 2	32	32	128	0.25
9	TA	> 32	> 1	> 8	≤ 2	> 8	> 2	32	32	32	0.25
10	TA	> 32	> 1	> 8	≤ 8	> 8	> 2	32	32	128	0.25
11	Blood	> 32	> 1	> 8	≤ 8	> 8	> 2	32	32	128	0.25
12	TA	> 32	> 1	> 8	≤ 8	> 8	> 2	32	32	128	0.25
13	TA	> 32	> 1	> 8	≤ 8	> 8	> 2	32	32	128	64
14	Blood	> 32	> 1	> 8	≤ 2	> 8	> 2	32	32	128	1
15	TA	> 32	> 1	> 8	≤ 2	> 8	> 2	32	32	128	0.25
16	TA	> 32	> 1	> 8	≤ 8	> 8	> 2	32	32	128	0.25
17	TA	> 32	> 1	> 8	≤ 4	> 8	> 2	32	32	128	0.5
18	TA	> 32	> 1	> 8	≤ 2	> 8	> 2	32	32	128	0.25
19	TA	> 32	> 1	> 8	≤ 2	> 8	> 2	32	32	256	2
20	Blood	16	> 1	> 8	≤ 8	> 8	> 2	32	32	256	128
21	TA	> 32	> 1	> 8	≤ 2	> 8	> 2	32	32	512	0.25

*TA* tracheal aspiration, *MIC*, minimal inhibitory concentration, *AK* amikacin, *CIP* ciprofloxacin, *CN* gentamicin, *SXT* trimethoprim–sulfamethoxazole, *IMP* imipenem, *LEV* levofloxacin, *MEM* meropenem, *ETP* ertapenem, *FF* fosfomycin, *CT* colistin

### Synergy assay results

#### Checkerboard synergy assay results

The results of the checkerboard synergy analysis of the CRAB isolates are shown in Table 3. All the isolates showed a synergistic interaction for the CT–MEM combination. In addition, 52.3% of the isolates showed a synergistic interaction for the MEM–FF combination. The synergy rates for MEM–ETP and CT–FF were 23.8% and 28.5%, respectively. Among all combinations that were analyzed, antagonism was detected in two patients. One of these patients was in the MEM–ETP combination group, and the other patient was in the CT–FF combination group.

**Table 3:** In vitro synergy testing against *A. baumannii* by checkerboard and *E* test methodology, treatment regimen, clinical outcome, presence of death due to infection and, if any, on which day of treatment

	Checkerboard assays				*E* test synergy assays							
No	CT–MEM	CT–FF	MEM–ETP	MEM–FF	CT–MEM	CT–FF	MEM–ETP	MEM–FF	Treatment Regimen	Clinical outcome	Infection-related death	Death on which day of treatment
1	S	I	S	Add	Ant	Ant	I	Ant	MEM–CT	TF	+	3
2	S	Add	S	Add	Ant	Ant	I	Ant	MEM–CT	TF	+	8
3	S	Add	Ant	Add	Ant	Ant	I	I	MEM–CT	ME	-	-
4	S	I	I	Add	I	I	I	Ant	MEM–CT	ME	-	–
5	S	Add	I	Add	Ant	Ant	I	Ant	MEM–CT	TF	+	22
6	S	S	I	S	Ant	Ant	I	I	MEM–CT	ME	–	–
7	S	Add	I	S	Ant	Ant	I	I	MEM–CT	ME	–	–
8	S	I	I	S	Ant	Ant	I	I	MEM–CT	TF	+	45
9	S	Ant	I	I	Ant	Ant	I	Ant	MEM–CT	ME	–	–
10	S	I	I	I	Ant	Ant	I	Ant	MEM–CT	TF	+	4
11	S	Add	Add	Add	Ant	Ant	I	Ant	MEM–CT	TF	+	12
12	S	I	Add	Add	Ant	Ant	I	Ant	MEM–CT	ME	–	–
13	S	S	Add	S	S	S	I	Ant	MEM–CT	ME	–	–
14	S	Add	Add	S	I	I	I	Ant	MEM–CT–FF	ME	–	–
15	S	I	Add	Add	Ant	Ant	I	Ant	MEM–CT	C	–	–
16	S	S	Add	S	Ant	Ant	I	Ant	MEM–CT	ME	–	–
17	S	I	Add	S	Ant	Ant	I	Ant	MEM–CT–TGS	ME	–	–
18	S	Add	S	S	Ant	Ant	I	Ant	MEM–CT	ME	–	–
19	S	S	Add	S	I	I	I	I	MEM–CT–SXT	ME	–	–
20	S	S	S	S	S	S	I	I	MEM–CT–FF–VAN	TF	+	6
21	S	S	S	S	Ant	Ant	I	I	MEM–CT	C	–	-

The interactions between the 2 drugs were determined according to:

*S* Synergy, *Add* Additivity: *I* Indifference: *Ant* Antagonism: *MEM* meropenem; *CT* colistin: *ETP* ertapenem: *FF* fosfomycin: *TGS* tigecycline; *SXT* trimethoprim–sulfamethoxazole; *VAN* vancomycin; *ME* microbial eradication; *TF* treatment failure, *C*, cure

#### E synergy assay results

All isolates showed indifference in interaction for the MEM–ETP combination. Seven isolates showed indifference interaction with the MEM–FF combination, while the other isolates showed antagonistic interactions. Two isolates had synergistic effects, three isolates had indifference effects, and the other isolates had antagonistic effects for the CT–MEM and CT–FF combinations (Table 3).

In vitro synergy was demonstrated in 43 (51.1%) of 84 possible isolate/antibiotic combinations by checkerboard methods. However, a synergistic effect was detected in only four isolates via the *E* test method. For the checkerboard/*E* test method, the additive, indifferent and antagonistic effects were determined to be 23/0, 16/34, and 2/46, respectively, of the possible 84 isolate/antibiotic combinations. The same test outcome between the two methods was reported for 11 (6.5%) of the 168 possible isolate/antibiotic combinations (Table 3).

### Clinical outcomes

All of the patients were treated with CT–MEM-based combinations, and microbiological eradication was achieved in twelve (57.1%) patients. MEM–CT combination therapy was used in the treatment of nine of the patients who achieved microbial eradication. In addition to MEM–CT treatment in the other three patients, FF was used in one patient, tigecycline was used in one patient, and trimethoprim/sulfamethoxazole (SXT) was used in one patient. Microbial eradication was achieved in 52.9% (9/17) of patients receiving only CT–MEM combination therapy. Although a cure was achieved without microbial eradication in two (9.5%) patients, treatment failure occurred in seven (33.3%) patients. MEM–CT–FF–vancomycin combination therapy was used in the treatment of one of the patients with treatment failure. All patients who experienced treatment failure died due to infection (Table 3).

## Discussion

CT shows excellent antibacterial activity in the treatment of infections caused by CRAB isolates [19]. However, the efficacy of colistin monotherapy has been questioned because of its low plasma concentrations, heteroresistance and rapid posttreatment growth [19]. Therefore, combination therapy is preferred for the treatment of CRAB infections [20]. MEM, a carbapenem antibiotic, has a low toxicity profile and is resistant to many serine–lactamases produced by multidrug-resistant (MDR) gram-negative bacteria, thus playing a key role in combination therapy for CRAB infections [21]. The combination of CT and MEM is the most commonly preferred antimicrobial combination for the treatment of these infections [1]. In this combination, CT potentiates the activity of carbapenems through depolarization of the outer cell membrane, allowing carbapenems greater access to their target sites within the periplasmic space [1]. In our study, all *A. baumannii* isolates analyzed by the checkerboard method showed a synergistic effect on the CT–MEM combination. The CT–MEM synergy rates in *A. baumannii* isolates have been reported to range widely between 17.5% and 100% [22–24]. This large difference in the synergy percentage range may be related to the use of different synergy tests in the studies. Several methods, such as the time-kill assay, checkerboard and *E* test, are used for in vitro antimicrobial synergy testing [25]. It has been reported that the synergy rates determined by the time-kill method are greater than the synergy rates determined by the *E* test and checkerboard method [23, 26]. For this reason, the synergy method used must be taken into account when comparing synergy studies. The CT–MEM synergy rates for *A. baumannii* isolates have been reported to be in the range of 32–100% in studies using the checkerboard method [23, 26–29]. Although the same methods were utilized, the in vitro synergy rates were inconsistent. There could be four reasons for this: 1. Four different methods can be used to interpret the checkerboard method [29]. A study comparing these interpretation methods revealed that the interactions between the same antimicrobial combinations vary according to the interpretation method [29]. We interpreted our checkerboard results according to method 1 [29]. The interpretation methods used in other studies are not specified. 2. In isolates of *A. baumannii* that contain carbapenemases, carbapenemases released from the periplasmic space with the cell wall degraded by colistin can degrade the MEM structure [30]. In our study, the carbapenemase production status of the isolates was unknown. 3. Different exposures of isolates to antimicrobial agents may cause differences in response to synergy tests. Therefore, in vitro tests should be performed prior to in vivo use [31]. In our study, eight patients had previously been treated with carbapenem, and three patients had previously been treated with both carbapenem and colistin. 4. There may be differences in the genetic environments of isolates from different regions [31]. This finding may explain the difference in the in vitro synergy test results.

Although CT–MEM combination therapy was started in all patients included in the study, six patients (35.2%) died due to treatment failure. In a multicenter study conducted by Paul et al., the 28-day mortality rate associated with CT–MEM treatment in patients with CRAB infection was 52% [3]. In a multicenter study conducted by Kaye et al., this rate was reported to be 42% [32]. In vitro results are not always consistent with in vivo results [33]. Pharmacokinetic/pharmacodynamic (PK/PD) factors, including age, comorbidity, volume of drug distribution, drug elimination rate and kidney and liver functions, are known to affect in vivo results [33]. We know that PK/PD studies go beyond evaluating in vitro parameters, such as MIC, minimal bactericidal concentration and mutant inhibitor concentration, in determining in vivo antimicrobial activities. Determination of synergistic activity alone does not guarantee therapeutic efficacy. However, due to the limitations of in vivo studies and the desperation for treatment, in vitro synergy studies are important for providing clinicians with ideas about treatment.

In a study by Lertsrisatit et al., the synergistic effect of the CT–MEM combination was 16.7%, and the mortality rate for colistin-resistant *A. baumannii* isolates was 70.6% [17]. Qureshi et al. reported a mortality rate of 30% in CT-resistant *A. baumannii* isolates [34]. In our study, one of the two CT-resistant isolates died due to treatment failure.

The CT–MEM synergy rate has been reported to be in the range of 16.7–96% in CT-resistant isolates [17, 22, 25, 27]. Both of the CT-resistant isolates included in our study showed synergistic effects on the CT–MEM combination. Interestingly, synergy rates were reported to be greater for CT-resistant isolates than for CT-sensitive isolates [22]. Hypothetically, colistin-resistant *A. baumannii* may have a modified outer membrane, which can increase permeability with respect to cell wall-targeted antimicrobial agents. However, the underlying mechanism is not fully known [27].

SXT is an antimicrobial agent that has been in use for over 40 years. SXT acts by inhibiting bacterial DNA synthesis through inhibition of the dihydrofolate pathway. SXT has good antibacterial activity against a broad spectrum of gram-positive and gram-negative bacteria. In current medical practice, SXT has not been recommended for the treatment of MDR *Acinetobacter* infections [35]. A review of 26 studies examining SXT resistance in CRAB isolates was conducted; in 22 of the studies, the SXT resistance rate was reported to exceed 80% [35]. It appears that nearly half of the isolates included in our study are sensitive to SXT. However, it appears that SXT is added to CT and MEM combination therapy in the treatment of only one patient. The risks to the patients included in the study are high. Therefore, empirical and specific antibiotic regimens were selected based on current literature data [36, 37]. Could SXT be an alternative antibiotic in the treatment of CRAB infections? Large-scale in vitro and in vivo studies are needed to answer the question.

Various studies have shown that dual carbapenem combinations have in vitro synergistic effects on carbapenem-resistant gram-negative bacterial infections [38]. The combination of ETP with another carbapenem was prompted by the evidence that ETP, as a suicide antibiotic, could bind to the active site of carbapenemase with high affinity, which further prevented the hydrolysis of the other carbapenem molecule and preserved its bactericidal activity [38]. In our study, the combination of the checkerboard method and MEM–ETP had synergistic effects on five patients. As we previously mentioned, the carbapenemase production of the isolates is unknown.

Recently, FF, an ''old'' drug, was introduced as a new option for the treatment of MDR *A. baumannii* infection [19]. Although FF is an active antimicrobial against gram-positive and gram-negative bacteria, the number of studies on its synergistic effect, especially in MDR *A. baumannii* isolates, is quite limited [19]. In a study by Ku et al., the CT–FF combination had a synergistic effect and reduced the bacterial load in the lungs within 24–48 h in a pneumonia mouse model caused by MDR *A. baumannii* isolates [19]. In another study of combination therapy against *A. baumannii*, colistin combined with FF was more effective than colistin monotherapy in MDR strains [39]. Tharavichitkul et al. reported that the CT–FF combination had a synergistic effect on CRAB [40]. Consistent with our study, we found a synergistic effect of 28.5% with the CT–FF combination.

In our study, a 52.3% synergistic effect was demonstrated with the MEM–FF combination. In their study, Adaleti et al. reported a synergistic effect for the MEM–FF combination in only one of six CRAB isolates [41]. Although it has been previously reported that this combination has a synergistic effect on *Pseudomonas aeruginosa* and *Klebsiella pneumoniae* isolates [42], the number of studies investigating the synergistic effect of this antimicrobial combination on CRAB isolates is quite limited in the current literature. Although MEM–CT-based antimicrobial combinations are frequently used to treat CRAB infections, increasing resistance rates make it necessary to investigate alternatives to this combination therapy. Therefore, we found this rate to be quite remarkable.

In one study, it was reported that the additive effect rate was significantly greater for the checkerboard method than for the *E* test method [30]. Our results were consistent with this study. In addition, in our study, synergistic effects were detected via the checkerboard method, and antagonistic effects were detected at a greater rate via the *E* test method. One study reported 63% agreement between the *E* test and the checkerboard test [43], which was higher than that of our study. A possible explanation for this inconsistency in the *E* test and checkerboard test techniques may be the difference in properties between the liquid media and solid media used in these experiments. Since colistin cannot be adequately dispersed in solid media, the EUCAST guidelines recommended the liquid microdilution method as the reference method in determining the MIC value of colistin [16]. Therefore, the incompatibility between the E synergy test techniques and the checkerboard test techniques in combinations with colistin may be attributed to a lack of diffusion in the solid medium. In our study, as explained in the methods section, before performing the *E* synergy test, it was checked whether the antibiotic from the *E* test strip had sufficiently diffused into the agar by using the standard strain. Moreover, there is no significant difference in terms of the compatibility of the two tests in combinations with and without colistin (compatibility between the results of the *E* synergy test and those of the checkerboard test was detected in 2, 4, 7 and 0 isolates in CT–MEM, CT–FF, MEM–ETP and MEM–FF combinations, respectively). Therefore, the difference between the *E* synergy test results and the checkerboard test results is not attributed to the lack of diffusion in the solid medium. To make more comprehensive comments on this subject, it is necessary to investigate the existence of synergy with a third method, such as time-kill.

Other limitations of our study are listed as follows: 1. Only 21 isolates were included in the study. Studies with larger samples are needed. 2. In our hospital, CT–MEM-based combined antibiotic therapy is mostly administered. For this reason, no comment could be made on the treatment results of the MEM–FF combination, for which we found a high synergy rate. 3. The carbapenem resistance mechanisms and clonal relationships of the isolates are unknown.

## Conclusions

A synergistic effect was found with the checkerboard method for the CT–MEM combination in all isolates in our study, and approximately 70% of the patients benefited from treatment with this combination. In addition, more than half of the isolates showed a synergistic effect for the MEM–FF combination. Combinations of CT–MEM and MEM–FF may be options for the treatment of CRAB infections. However, a comprehensive understanding of the potential of the microorganism to develop resistant mutants under applied exposures, as well as factors that directly affect antimicrobial activity, such as PK/PD, is essential for providing treatment advice. More comprehensive studies are needed on this subject. In addition, in our study, in contrast to the literature, a low rate of agreement between the *E* test method and the checkerboard test method was found. Treatment of CRAB infections, which is one of the most important problems of our age, is mostly performed with antibiotic combination therapy. However, in vitro synergy tests, which are one of the most important bases for antibiotic combination selection, lack standardization at every stage from the selection of the test technique to the interpretation of the tests. Comprehensive studies that compare clinical results with methods are needed to determine the ideal synergy test and interpretation method.

## Data Availability

No datasets were generated or analysed during the current study.

## Abbreviations

CRAB: Carbapenem-resistant *Acinetobacter baumannii*
CT: Colistin
MEM: Meropenem
ETP: Ertapenem
FF: Fosfomycin
SXT: Trimethoprim–sulfamethoxazole
E: Epsilometer
MICs: Minimum inhibitory concentrations
FICI: Fractional inhibitory concentration index
MDR: Multidrug resistant
PK/PD: Pharmacokinetic/pharmacodynamic factors
